# Protein Methylation as a Regulatory Logic Layer in Cancer Signaling: Interplay with Phosphorylation and Network Plasticity

**DOI:** 10.3390/cancers18060903

**Published:** 2026-03-11

**Authors:** Kyung-Hee Kim, Byong Chul Yoo

**Affiliations:** 1 Department of Applied Chemistry, School of Science and Technology, Kookmin University, Seoul 02707, Republic of Korea; kyungheekim@kookmin.ac.kr; 2 Antibody Research Institute, Kookmin University, Seoul 02707, Republic of Korea; 3 Diagnostic Research Team, InnoBation Bio R&D Center, Seoul 03929, Republic of Korea

**Keywords:** protein methylation, non-histone methylation, cancer signaling, post-translational modification, signal transduction, network plasticity, therapeutic resistance, phosphorylation crosstalk

## Abstract

Phosphorylation has long been regarded as the principal mechanism governing oncogenic signal transduction. However, it does not fully account for the diversity, persistence, and context dependence of cancer signaling outputs. Protein methylation, historically studied in the context of histone regulation, is now recognized as a widespread modification of non-histone signaling proteins, including transcription factors, DNA damage response mediators, and scaffold components. In this Review, we propose that protein methylation functions as a regulatory logic layer that shapes how oncogenic signals are amplified, stabilized, and interpreted. Rather than serving as a primary trigger of pathway activation, methylation modulates signaling behavior across four interconnected dimensions: activation threshold and signal gain, temporal persistence, network topology and complex assembly, and spatial routing. We examine major signaling axes in which methylation refines genome integrity networks, proliferative pathways, inflammatory circuits, and lineage-specific transcriptional programs. We further discuss the interdependency between methylation and phosphorylation, highlighting sequential, competitive, and feedback-mediated interactions that expand combinatorial signaling states. Finally, we explore how methylation-mediated regulatory logic contributes to signaling plasticity and adaptive resistance under therapeutic pressure, and we outline key measurement and translational challenges. Framing protein methylation within a regulatory logic paradigm provides a structured approach for integrating this modification into contemporary models of oncogenic signaling and therapeutic intervention.

## Simple Summary

Cancer signaling has traditionally been viewed through a kinase-centered framework in which phosphorylation acts as the primary driver of pathway activation. However, phosphorylation alone does not fully explain the diversity, persistence, and adaptability of signaling observed in tumors. This review highlights protein methylation as an additional regulatory layer that refines how oncogenic signals are interpreted and sustained. Unlike phosphorylation, which often acts as an on–off switch, methylation can influence signaling thresholds, stability, complex assembly, and spatial organization of key proteins. Through its interplay with phosphorylation, methylation expands the range of possible signaling states and may contribute to therapeutic resistance. We discuss major signaling pathways in which methylation modifies cancer cell behavior and examine emerging efforts to target methylation regulators in clinical settings. Understanding methylation as a regulatory logic layer may provide new opportunities for combination therapies and more durable control of cancer signaling networks.

## 1. Introduction

Cancer signaling has long been interpreted through a kinase-centric framework in which phosphorylation functions as a primary driver of signal initiation and propagation [1,2]. This perspective has yielded deep mechanistic insights into oncogenic activation and pathway hierarchies and has enabled major therapeutic advances, particularly for receptor tyrosine kinases, PI3K–AKT–mTOR signaling, and MAPK cascades [3,4,5]. The clinical success of kinase inhibitors across multiple cancer types further reinforces the centrality of phosphorylation in tumor biology [6,7,8].

At the same time, accumulating evidence indicates that phosphorylation alone does not fully account for the diversity, persistence, and context dependence of oncogenic signaling outputs. Similar upstream kinase activation can produce markedly different transcriptional programs, cell-state transitions, and therapeutic responses depending on cellular lineage, metabolic conditions, and microenvironmental cues [9,10,11,12]. In many settings, signaling amplitude does not translate linearly into biological outcome, and transient pathway activation can yield durable phenotypic effects [13,14]. These features point to additional regulatory layers that shape how signaling information is interpreted and executed in cancer cells.

Protein methylation has emerged as one such layer. While historically linked to histone regulation and epigenetics, methylation is now recognized to occur broadly on non-histone proteins, including transcription factors, signaling intermediates, DNA damage response components, and scaffold proteins [15,16]. For example, Set7-dependent methylation of YAP provides a representative case in which non-histone methylation directly intersects with a core oncogenic signaling module (Hippo/YAP), shaping pathway output through altered YAP regulation [17]. Both lysine and arginine residues can undergo mono-, di-, or tri-methylation, catalyzed by distinct methyltransferase families and, in many cases, reversed by demethylases [18,19]. Crucially, these modifications exert direct effects on protein stability, interaction interfaces, subcellular localization, and activity, rather than merely recapitulating histone-associated mechanisms [20,21].

**Figure 1:**
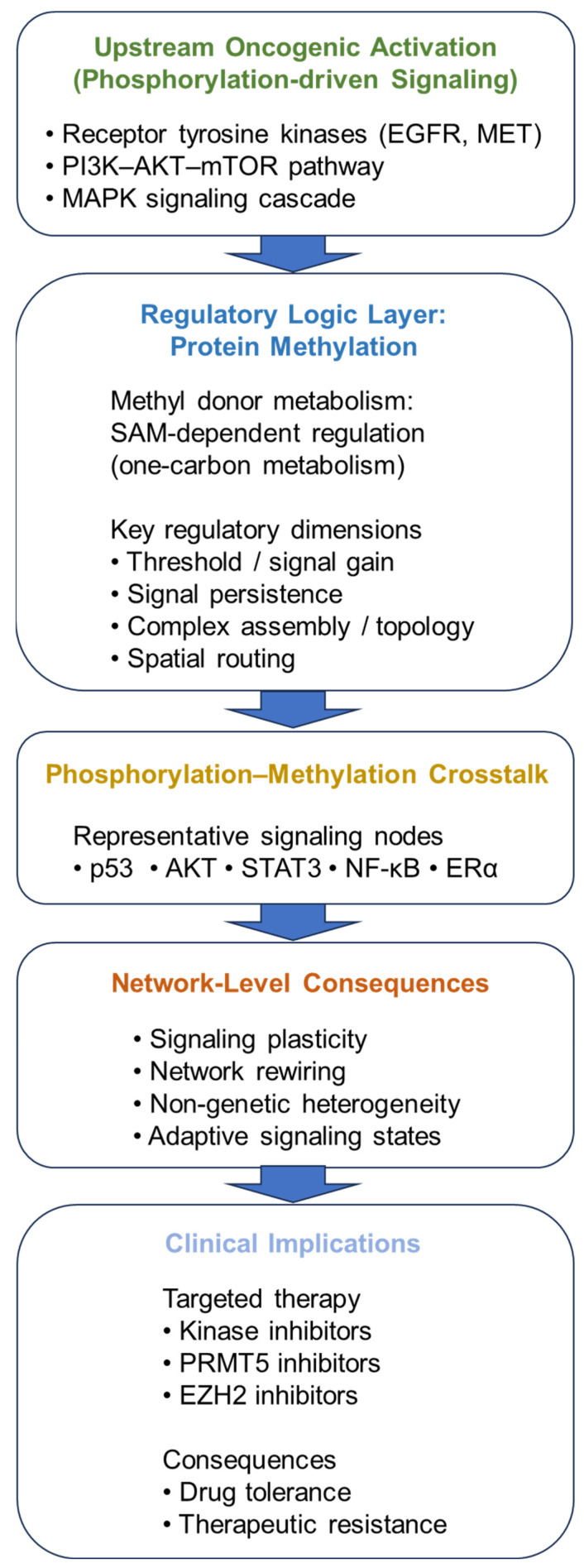
Integrated model of phosphorylation–methylation regulatory logic in oncogenic signaling. Canonical oncogenic signaling is primarily initiated by phosphorylation-driven pathway activation through receptor tyrosine kinases and downstream signaling cascades such as PI3K–AKT–mTOR and MAPK pathways. Protein methylation functions as a regulatory logic layer that modulates signaling behavior rather than initiating activation. Because methylation reactions depend on S-adenosylmethionine (SAM), methylation capacity is metabolically coupled to one-carbon metabolism. Through interdependency with phosphorylation, methylation influences key regulatory dimensions including activation threshold, signal persistence, complex assembly, and spatial routing. Representative signaling proteins involved in phosphorylation–methylation crosstalk include p53, AKT, STAT3, NF-κB, and ERα. These layered regulatory interactions reshape signaling networks and promote signaling plasticity, network rewiring, and non-genetic heterogeneity. At the clinical level, these dynamics contribute to adaptive responses to targeted therapies, including kinase inhibitors and methylation-targeted agents such as PRMT5 and EZH2 inhibitors. Representative disease contexts include EGFR-driven lung cancer, BRAF-mutant melanoma, and ERα-positive breast cancer, in which phosphorylation–methylation regulatory logic may shape therapeutic adaptation.

Because methylation reactions depend on S-adenosylmethionine (SAM), methylation capacity is coupled to cellular metabolic state, adding a context-dependent layer to signaling regulation [18,19]. This metabolic coupling provides a biochemical foundation for the regulatory logic layer illustrated in Figure 1, linking methyl donor availability to context-dependent signaling behavior within oncogenic signaling networks [18,19]. Compared with phosphorylation, which often introduces strong electrostatic changes that can rapidly switch protein states, methylation typically alters side-chain chemistry more subtly and without major charge alteration [2,22]. Through these effects, methylation can influence protein–protein interactions, accessibility of neighboring modification sites, and stability of multiprotein complexes, thereby shaping signaling thresholds, persistence, and network topology rather than acting primarily as an on–off switch [12,23].

In cancer, non-histone protein methylation has been implicated in regulation of tumor suppressor networks, oncogenic signaling hubs, inflammatory pathways, and lineage-specific transcriptional programs [16,20,24]. Methylation of p53, for instance, has been reported to influence transcriptional selectivity and stability, thereby biasing cell fate decisions under stress [25,26,27,28]. Methylation events within growth and inflammatory signaling axes have similarly been linked to altered pathway persistence and downstream transcriptional output [29,30,31]. Collectively, these observations suggest that methylation contributes to signaling behavior as a contextual determinant of output.

Importantly, methylation frequently operates in concert with other post-translational modifications. Interdependency with phosphorylation can occur through sequential modification of neighboring residues, competitive site occupancy, or indirect regulation of modifying enzymes [30,32,33]. Such interactions increase the combinatorial complexity of signaling networks and may contribute to the robustness and plasticity characteristic of cancer cells [12,34,35]. Rather than displacing phosphorylation, methylation appears to refine kinase-driven signaling, enabling tumors to rewire pathway outputs under selective pressures, including targeted therapy [7,36].

These insights raise translational questions. How does protein methylation influence signaling persistence and adaptive resistance to kinase inhibitors? Can methylation states serve as biomarkers of pathway dependency or therapeutic vulnerability? And under which contexts might pharmacologic modulation of methyltransferases reshape oncogenic signaling networks in clinically meaningful ways [24,37,38]? Although inhibitors targeting selected methylation regulators are advancing into clinical development [39,40,41,42,43], a coherent framework linking methylation-mediated regulation to signaling logic and therapy response remains incomplete [24].

In this Review, we examine protein methylation as a regulatory layer that contributes to the rewiring of cancer signaling networks. In particular, this review proposes a conceptual framework in which protein methylation functions as a regulatory “logic layer” that shapes signaling thresholds, persistence, and network topology within oncogenic signaling networks. Focusing on non-histone substrates, we first outline functional consequences of methylation for signaling proteins, emphasizing stability control, complex assembly, and spatial organization. We then analyze major signaling axes where methylation recurrently modulates pathway behavior and discuss interdependency with phosphorylation and other PTMs. Finally, we consider implications for signaling plasticity and therapeutic adaptation and highlight key technical and translational challenges. By organizing available evidence around regulatory logic rather than enzyme families alone, we propose that protein methylation functions as a contextual layer that shapes how oncogenic signals are amplified, stabilized, and reinterpreted across cancer signaling networks. Beyond mechanistic insight, understanding methylation-dependent regulatory logic may inform rational therapeutic combinations and biomarker-driven patient stratification. Although this Review emphasizes methyltransferase-driven regulation, lysine demethylases (e.g., KDM family members) and related regulatory enzymes further modulate methylation dynamics, contributing to context-specific signaling outputs [18,21].

## 2. The Regulatory Logic of Protein Methylation in Cancer Signaling

Cancer signaling is frequently framed in terms of pathway “activation” or “inhibition,” with phosphorylation as the principal driver of information transfer [1]. However, signaling outputs depend not only on activation status but also on magnitude, duration, spatial routing, and modification context [11,12,13,14]. Protein methylation contributes to these regulatory dimensions by shaping how phosphorylation-driven signals are amplified, stabilized, and interpreted. This conceptual framework is summarized in Figure 1 [15].

### 2.1. Threshold Setting and Signal Gain Control

Cells must discriminate between basal noise and meaningful stimulation, establishing activation thresholds that prevent inappropriate responses [11,14]. Protein methylation can adjust this threshold by altering interaction affinity, complex recruitment, or transcriptional competence of downstream effectors [15,16]. In many contexts, methylation does not initiate signaling but modulates the efficiency with which a phosphorylation-driven cue is converted into downstream output. Unlike phosphorylation, which can directly activate signaling cascades through large electrostatic and conformational changes, methylation typically introduces more subtle structural modifications that influence protein interactions or stability [22,23]. Consequently, methylation more often functions as a contextual regulator of signaling efficiency rather than a primary oncogenic driver [12,15]. For example, methylation of transcription factors or co-regulators can influence cofactor recruitment and transcriptional selectivity, modifying response strength under similar upstream kinase activity [25,26,27,28]. Such gain control may contribute to oncogenesis by lowering the effective threshold for growth and survival programs or, conversely, by raising thresholds for tumor suppressive responses such as arrest or apoptosis [20].

### 2.2. Signal Duration, Persistence, and “Memory”

Whereas phosphorylation is often highly dynamic, methylation can generate more persistent structural and interaction changes that prolong signaling consequences [13,15,30,44]. By stabilizing multiprotein assemblies, modulating protein turnover, or sustaining transcriptional programs after upstream activation declines, methylation introduces temporal decoupling between transient signaling events and durable cellular phenotypes [15,20,44].

### 2.3. Control of Complex Assembly and Network Topology

Methylation frequently occurs within or near interaction domains, where it can remodel binding interfaces and reconfigure signaling complexes [15,33,45]. By strengthening or weakening selective interactions, methylation may redirect pathway flux, bias branch selection, or reshape feedback connectivity—features central to network robustness and therapeutic resistance [7,34,35,36].

### 2.4. Spatial Regulation and Subcellular Routing

Signaling specificity also depends on spatial organization. Subcellular localization governs access to substrates, regulators, and DNA targets, and compartmentalized signaling can produce distinct outputs even with similar phosphorylation states [14]. Methylation can influence nuclear–cytoplasmic trafficking, membrane recruitment, or retention within specific compartments by modifying localization determinants or interaction partners [15,46,47]. Spatial redistribution can indirectly reshape phosphorylation dynamics by altering exposure to kinases and phosphatases, while also changing the downstream transcriptional context [30,34]. In oncogenesis, where mislocalization often contributes to aberrant signaling, methylation-dependent routing provides another mechanism by which signaling behavior may be tuned. In several cases, lysine or arginine methylation has been shown to influence nuclear import or export by modifying interaction surfaces recognized by transport factors such as importins or exportins [15,46]. Such methylation-dependent localization shifts can redirect signaling proteins between cytoplasmic signaling complexes and nuclear transcriptional programs, thereby altering pathway interpretation without necessarily changing upstream kinase activation [47].

Together, these principles position protein methylation as a contextual regulator of signaling outputs (Table 1), providing a conceptual framework through which phosphorylation-driven pathway activation can generate diverse signaling behaviors depending on modification context. Rather than acting as a primary trigger, methylation shapes thresholds, persistence, assembly, and routing—the features that largely determine whether phosphorylation-driven activation is interpreted as transient stress, durable proliferation, or adaptive survival. The following sections apply this framework to major signaling axes in cancer and then examine how methylation–phosphorylation interdependency contributes to network plasticity and therapeutic adaptation.

## 3. Signaling Axes Where Methylation Shapes Oncogenic Behavior

Within the conceptual framework proposed in this review, protein methylation exerts context-dependent regulatory effects across multiple oncogenic signaling axes. Rather than functioning as a universal activator or suppressor, methylation modifies how signaling networks behave under defined cellular conditions. Below, we examine major domains in which methylation-mediated modulation has been recurrently observed, emphasizing functional consequences over enzyme-specific cataloging [16].

### 3.1. Genome Integrity and Cell Cycle Control

Distinct lysine methylation events influence p53 stability, cofactor interactions, and promoter selectivity, thereby biasing stress responses toward arrest, apoptosis, or adaptive survival under comparable upstream signals [25,26,27,28]. Mechanistically, these methylation events can alter recruitment of transcriptional cofactors and chromatin-associated regulators, thereby influencing the selection of specific p53 target promoters that govern distinct cellular outcomes such as cell-cycle arrest or apoptosis [25,26,27,28]. Rather than functioning as binary switches, these modifications fine-tune transcriptional output within tumor suppressor networks [20]. Methylation-dependent regulation of additional cell-cycle mediators, including RB family proteins, further illustrates how combinatorial PTM states influence proliferation checkpoints [33,48].

### 3.2. Survival and Growth Signaling Pathways

Within proliferative pathways, methylation of key intermediates can alter stability, localization, or feedback sensitivity, thereby sustaining downstream transcriptional output without necessarily increasing upstream phosphorylation [20]. Consistent with this concept, oncogenic methyltransferases such as SMYD3 have been shown to promote cancer cell proliferation, supporting a direct link between methylation capacity and growth-program maintenance [49]. Such modulation enhances signaling robustness under fluctuating activation conditions [34]. For example, methylation-dependent regulation within PI3K–AKT signaling and metabolic integration networks underscores how methylation reinforces survival signaling under metabolic stress or therapeutic inhibition [5,44]. In addition to altered expression, several methylation regulators are recurrently mutated or genomically dysregulated in human cancers, including enzymes such as EZH2 and SETD2, further supporting the pathological relevance of methylation-dependent signaling regulation [20,24].

### 3.3. Inflammatory and Immune Signaling Networks

In inflammatory networks such as NF-κB and STAT signaling, methylation refines transcriptional selectivity and signal persistence independently of upstream phosphorylation amplitude [29]. By stabilizing inflammatory gene programs, these modifications contribute to tumor–immune adaptation and signaling plasticity [35]. Mechanistic studies demonstrate methylation-dependent regulation of RelA stability and STAT3 transcriptional activity [31,50], while broader reviews position these pathways at the interface of inflammation and oncogenesis [51,52]. Notably, arginine methylation of STAT family proteins has been discussed for years, underscoring that STAT signaling output can be tuned by methylation-dependent mechanisms beyond phosphorylation alone [53].

Beyond tumor cell–intrinsic signaling, methylation-dependent regulation of NF-κB and STAT pathways may influence the tumor immune microenvironment. Because these transcriptional programs govern cytokine production, antigen presentation, and immune checkpoint ligand expression, methylation-mediated stabilization of inflammatory signaling could shape antitumor immunity. Modulation of arginine methyltransferases has been reported to alter immune cell differentiation and T-cell effector function, suggesting that methylation inhibitors may exert both direct tumor cell–intrinsic effects and indirect immunomodulatory consequences [20,38,52].

### 3.4. Hormone and Lineage-Specific Signaling Programs

Estrogen receptor α (ERα) is a central lineage-defining driver in a substantial fraction of breast cancers, and its activity state critically shapes tumor phenotype and endocrine responsiveness [54]. In hormone-driven malignancies, methylation of nuclear receptors and associated cofactors influences receptor stability, complex assembly, and lineage-specific transcriptional routing [46]. These effects may alter hormonal responsiveness and contribute to phenotypic heterogeneity and therapy resistance [20,35]. SET7-dependent methylation of ERα and related cofactors provides molecular support for this regulatory axis [47], and metabolic rewiring linked to endocrine resistance further contextualizes lineage-specific signaling adaptation [55].

## 4. Interdependency Between Methylation and Phosphorylation

These interdependent modification patterns are not merely conceptual constructs but are increasingly supported by site-specific mechanistic evidence, several representative examples of which are summarized in Table 2 [32]. Representative examples summarized in Table 2 illustrate how phosphorylation of defined residues—such as p53 Ser15, RelA Ser536, AKT Thr308/Ser473, ERα Ser118, or STAT3 Tyr705—coexists with lysine or arginine methylation on the same protein to generate combinatorial regulatory states [25,26,29,30,31,46]. Importantly, these modifications often occur in spatially distinct domains, enabling methylation to modulate stability, complex assembly, or transcriptional selectivity without necessarily altering the initiating phosphorylation event [15].

Such layered regulation expands signaling plasticity by introducing alternative functional outcomes under similar upstream kinase activation [11,12,35]. Rather than functioning as redundant modifications, phosphorylation and methylation frequently establish hierarchical, cooperative, or competitive relationships that reshape network behavior [32,33]. In this framework, phosphorylation largely determines signal trajectory, whereas methylation frequently refines signal persistence, interpretive context, and resistance potential [15]. These site-specific combinatorial PTM states provide a molecular substrate for adaptive signaling under therapeutic pressure (Table 2) [7,35,36].

The combinatorial interplay between phosphorylation and methylation may also contribute to inter-cellular signaling heterogeneity within tumors. Because methylation states can differ in abundance, stoichiometry, and persistence across genetically similar cells, they introduce additional layers of non-genetic regulatory variability. Even in the absence of new mutations, differential methylation-dependent stabilization of signaling intermediates may bias pathway outputs toward distinct transcriptional states. In this framework, protein methylation expands the functional signaling landscape beyond binary on–off kinase activation, generating diverse interpretive states under comparable upstream inputs [12,35,56].

**Table 2:** Site-Specific Phosphorylation–Methylation Interdependency and Therapeutic Resistance in Cancer Signaling.

Upstream Kinase	Phosphorylated Protein (Phospho Site)	Methyltransferase (Methylation Site)	Mode of Interdependency	Functional Consequence	Therapeutic Resistance Relevance	Key Reference
ATM	p53 (Ser15)	SET7/9 (Lys372); SMYD2 (Lys370); SET8 (Lys382)	Cooperative/Competitive	SET7/9 stabilizes p53; SMYD2 attenuates transcription; SET8 modulates activity	Alters stress bias under genotoxic therapy	[25,26,27,28]
IKKβ	RelA/p65 (Ser536)	PRMT1 (Arg30); SETD6 (Lys310)	Sequential/Feedback modulation	PRMT1 enhances transcription; SETD6 represses NF-κB	Sustained inflammatory signaling → therapy resistance	[29,50,57]
PDK1/mTORC2	AKT (Thr308/Ser473)	SETDB1 (Lys64)	Cooperative stabilization	Methylation enhances AKT stability and kinase activity	Buffers PI3K–AKT inhibition	[44]
MAPK/AKT	ERα (Ser118/Ser167)	SET7/9 (Lys302)	Sequential reinforcement	Stabilizes ERα and nuclear retention	Endocrine resistance	[46,47]
JAKs	STAT3 (Tyr705)	SET9; EZH2	Cooperative persistence	Sustains STAT3 transcriptional bias	JAK inhibitor resistance	[31,58]
CDK4/6	RB (CDK phospho sites)	SMYD2 (Lys810)	Competitive/Structural	Alters RB stability and E2F control	Adaptive response to CDK4/6 inhibitors	[33,48]

### 4.1. Sequential and Hierarchical Regulation

Phosphorylation can create structural or interaction contexts that enable subsequent methylation, while prior methylation may modulate kinase accessibility [30,33,34]. Such ordered modification events introduce temporal structure into signaling responses. An initial phosphorylation pulse may activate a pathway, whereas subsequent methylation stabilizes or refines the response [14,31,58]. Conversely, methylation may prime proteins for phosphorylation under defined cellular conditions, such as growth factor stimulation, metabolic stress, or DNA damage signaling [30,44].

### 4.2. Competitive and Structural Interactions

Methylation near phosphorylation sites can influence kinase binding, substrate recognition, or phosphatase accessibility [30,33,34]. Although methylation does not alter charge as dramatically as phosphorylation, steric and conformational effects may generate mutually exclusive modification states [22,23]. Such competitive interactions bias signaling outcomes and may contribute to heterogeneity within tumor populations [35,56].

In addition to influencing kinase accessibility, methylation may also modulate phosphatase recruitment or substrate recognition. Because phosphorylation status reflects the dynamic balance between kinase activity and phosphatase-mediated dephosphorylation, methylation-dependent remodeling of protein interfaces could indirectly regulate phosphatase access to specific residues. Although this area remains less extensively characterized than kinase–methylation interplay, emerging evidence suggests that post-translational crosstalk can influence both arms of the phosphorylation equilibrium, thereby reshaping signaling persistence and feedback strength [22,34].

### 4.3. Feedback Architecture and Persistence

Feedback loops are central to pathway stability [5,12,14]. Methylation-mediated stabilization of signaling mediators or modification of negative regulators can reshape feedback strength, indirectly influencing phosphorylation dynamics [30,44,57,58]. In therapeutic contexts, such interactions may sustain downstream transcriptional programs even when kinase activity is pharmacologically reduced [7,35,36]. Rather than restoring phosphorylation directly, methylation may preserve network connectivity and robustness.

The interplay between methylation and phosphorylation underscores that signaling outputs arise from combinatorial modification landscapes. Representative site-specific examples illustrating phosphorylation–methylation interdependency and their implications for therapeutic resistance are summarized in Table 2.

Together, these interdependent modification patterns suggest that phosphorylation determines the immediate trajectory of signal transmission, whereas methylation frequently influences the stability and interpretive context of that signal. This layered interdependency complicates linear pathway models and highlights the need for integrative analysis of combinatorial PTM states. Such layered regulation expands the dynamic range of oncogenic networks and likely contributes to their resilience under pharmacologic perturbation [7,11,12].

### 4.4. Methylation-Dependent Signaling Heterogeneity in Tumor Populations

Intratumoral heterogeneity represents a fundamental feature of cancer biology and therapy response, reflecting not only genetic diversification but also non-genetic regulatory variability among tumor cells [56]. Protein methylation may contribute to this diversity by modulating how signaling pathways are interpreted rather than simply altering pathway activation. Because methylation can influence protein stability, interaction interfaces, and transcriptional selectivity, differences in methylation states across tumor subpopulations may generate distinct signaling persistence profiles even under similar upstream kinase activation.

Unlike genetic mutations, which impose relatively stable pathway rewiring, methylation-dependent regulatory states can fluctuate dynamically in response to metabolic conditions, microenvironmental signals, or therapeutic pressure. As a result, protein methylation may expand the functional signaling landscape within tumors, enabling subsets of cells to adopt alternative transcriptional or signaling states without requiring new genetic alterations. Such regulatory variability may contribute to phenotypic plasticity and facilitate the emergence of drug-tolerant cell populations [35].

In this framework, protein methylation functions as a regulatory layer that increases the range of possible signaling outputs from a common phosphorylation-driven input. By introducing additional combinatorial regulatory states within signaling networks, methylation may contribute to the inter-cellular signaling heterogeneity observed in clinical cancer populations [12].

## 5. Signaling Plasticity and Therapeutic Adaptation

Whereas kinase inhibitors primarily attenuate upstream pathway activation, methylation-dependent regulation may sustain downstream signaling persistence even under partial kinase suppression, thereby providing a mechanistic basis for non-genetic adaptive resistance [7,24,35,59]. At the molecular level, representative site-specific examples summarized in Table 2 demonstrate how phosphorylation–methylation interdependency stabilizes signaling mediators such as AKT, ERα, NF-κB, or STAT3, thereby preserving downstream transcriptional programs despite attenuated upstream activation [29,30,31,44,46,58]. In this framework, Table 2 details representative combinatorial PTM states that mechanistically enable such buffering behavior [12,34]. These observations underscore that therapeutic resistance may arise not only from genetic alterations or pathway reactivation but also from methylation-dependent modulation of signaling persistence and topology [7,24,35,56,59].

### 5.1. Adaptive Resistance to Targeted Therapy

Resistance to kinase inhibitors frequently involves pathway reactivation, compensatory signaling, or transcriptional reprogramming [6,7,35,59,60]. A canonical example of bypass/compensatory rewiring is MET amplification, which can reactivate downstream survival signaling and drive resistance to EGFR inhibition [61]. Similarly, feedback relief can rapidly restore pathway flux under targeted therapy, as illustrated by EGFR feedback activation that undermines BRAF(V600E) inhibition in colorectal cancer [62]. In melanoma, resistance to BRAF(V600E) inhibitors can emerge through receptor tyrosine kinase upregulation or NRAS-driven reactivation, highlighting how network context—not only the inhibited kinase—determines escape trajectories [63]. Methylation-dependent stabilization of signaling mediators or transcription factors may preserve essential survival programs despite reduced phosphorylation [30,31,44,58]. Additionally, methylation-mediated alteration of scaffold assembly can facilitate alternative routing through parallel pathways [5,33,45]. These mechanisms support non-genetic resistance by reshaping network context rather than restoring the original oncogenic mutation. Molecular examples of such combinatorial PTM states are detailed in Table 2. Importantly, such adaptive responses do not necessarily require new genetic alterations. Heterogeneous methylation states across tumor subpopulations may generate diverse signaling persistence profiles, enabling subsets of cells to transiently tolerate kinase inhibition. This non-genetic signaling diversity can precede and potentially facilitate the emergence of stable resistance-conferring mutations, reinforcing the concept that methylation contributes to phenotypic plasticity within evolving tumor ecosystems [35].

### 5.2. Metabolic Coupling

Methyl donor availability links cellular metabolism to methylation capacity [18,19,64,65,66]. Changes in one-carbon metabolism, nutrient status, or mitochondrial function may influence methylation dynamics and thereby modulate signaling persistence [5,15,18]. Under stress conditions such as hypoxia, metabolic shifts could alter methylation-mediated stabilization of survival pathways [35,55]. Although mechanistic details remain under investigation, this metabolic coupling reinforces the role of methylation in adaptive regulation.

Because SAM availability depends on methionine cycle flux and one-carbon metabolism, metabolic rewiring can directly influence protein methylation dynamics [64,65,66]. This metabolic dependency introduces an additional regulatory layer through which nutrient status and mitochondrial function reshape signaling persistence in cancer cells [5,64].

Beyond global one-carbon flux, regulation of S-adenosylmethionine (SAM) synthesis itself may critically influence methylation-dependent signaling states [64,65,66]. SAM is generated by methionine adenosyltransferase (MAT) isoforms, including MAT1A and MAT2A, with MAT2B functioning as a regulatory subunit that modulates MAT2A activity. In cancer, a well-documented MAT1A-to-MAT2A isoform switch has been associated with altered methylation potential and tumor progression [64,65]. Dysregulated MAT2A expression or MAT2B-mediated modulation may influence intracellular SAM availability and thereby reshape protein methylation dynamics in a context-dependent manner. In parallel, lysine and arginine demethylases further contribute to reversibility and spatial specificity of methylation states [18,21], reinforcing that protein methylation reflects a dynamically regulated metabolic–enzymatic equilibrium rather than a static modification layer.

### 5.3. Rational Therapeutic Integration

Targeting methyltransferases is unlikely to replace kinase inhibition but may complement it in defined contexts [24,37,39,40]. This perspective aligns with the regulatory logic framework proposed in this review, in which methylation functions primarily as a stabilizing and interpretive layer rather than as the initiating trigger of oncogenic signaling. If methylation contributes to pathway persistence or feedback resilience, co-targeting methylation could reduce signaling robustness and delay resistance [7,35,36,44]. However, redundancy among enzymes and systemic effects necessitate careful mechanistic mapping and patient stratification [20,24,38]. In contrast to kinase inhibitors, which directly suppress pathway activation or signaling node activity, methylation-targeted therapies are more likely to modulate signal persistence, transcriptional reinforcement, and adaptive network robustness; accordingly, their greatest therapeutic value may lie in combination strategies rather than in stand-alone pathway suppression [24,35,59].

From a network perspective, methylation-mediated rewiring may occur through three principal mechanisms:

(i) stabilization of adaptive signaling states,
(ii) reconfiguration of interaction topology enabling alternative routing, and
(iii) reinforcement of transcriptional programs that sustain survival despite attenuated upstream activation.

These mechanisms do not require new genetic alterations but can emerge dynamically under therapeutic pressure [67,68]. This framework links methylation-dependent regulation directly to signaling plasticity in cancer.

### 5.4. Therapeutic Targeting of Protein Methylation

Although protein methylation has historically been studied within the context of chromatin regulation, pharmacologic targeting of methyltransferases has increasingly entered clinical development [24,37,39,40,42,43]. Among these, inhibitors of protein arginine methyltransferase 5 (PRMT5) represent the most advanced class, with several small molecules currently in early-phase clinical trials for solid tumors and hematologic malignancies [39,40,42,43]. Agents such as GSK3326595, JNJ-64619178, and PF-06939999 are being evaluated for safety, pharmacodynamic engagement, and potential combinatorial benefit (Table 3). Beyond PRMT5, pharmacologic inhibition of type I PRMTs has been enabled by cell-active chemical probes such as MS023, which provides a practical framework for testing how broad arginine methylation programs contribute to signaling reinforcement and therapy adaptation in cancer models [69].

Importantly, clinical development strategies rarely position methylation inhibitors as isolated signal suppressors. Rather, they are explored in combination with kinase inhibitors, DNA damage response-targeted agents, or immune checkpoint blockade [6,24]. This combination-oriented strategy aligns with the conceptual framework proposed in this Review: protein methylation does not typically function as the primary initiator of oncogenic signaling but instead modulates signal persistence, network topology, and transcriptional reinforcement.

Preclinical data further suggest that tumors characterized by specific vulnerabilities, such as MTAP deletion, may exhibit heightened sensitivity to PRMT5 inhibition [41,70], providing a potential biomarker-driven stratification strategy. Nevertheless, early-phase studies underscore challenges, including pathway redundancy, systemic toxicity, and incomplete suppression of adaptive signaling programs [20,24].

Taken together, therapeutic targeting of protein methylation is best conceptualized not as a parallel alternative to kinase inhibition, but as an intervention directed at the regulatory logic that underlies signaling plasticity. By disrupting methylation-dependent stabilization and network reconfiguration, combination strategies may attenuate the emergence of drug tolerance and adaptive resistance.

Whether sustained therapeutic benefit can be achieved without disrupting physiological methylation-dependent regulation remains an open clinical question.

## 6. Measurement Challenges and Translational Considerations

Despite increasing recognition of protein methylation as a regulator of oncogenic signaling, substantial technical and translational challenges limit its systematic clinical integration. Unlike genomic alterations or even phosphorylation-based readouts, non-histone protein methylation remains difficult to quantify with precision and reproducibility in clinical specimens.

### 6.1. Technical Limitations in Quantifying Protein Methylation

A primary challenge lies in the frequently low stoichiometry of non-histone methylation events. In contrast to phosphorylation, which often displays rapid and high-amplitude changes in response to extracellular stimuli, methylation modifications may occur on only a small fraction of the total protein pool [15,71]. This substoichiometric nature complicates detection and quantitative interpretation, particularly when attempting to distinguish functional regulatory states from background modification.

Site-specific quantification presents additional complexity. Lysine and arginine residues may exist in mono-, di-, or trimethylated forms, and these states are not always reliably discriminated by antibody-based methods [21,71]. Cross-reactivity, limited epitope accessibility, and dependence on protein conformation further constrain immunodetection strategies. The relatively subtle chemical difference introduced by lysine or arginine methylation may also reduce epitope antigenicity, making it difficult to generate highly specific antibodies capable of reliably distinguishing methylation states [21,71]. As a result, antibody-based assays may provide qualitative rather than rigorously quantitative information.

Mass spectrometry-based proteomics offers higher specificity and site resolution; however, comprehensive methylation profiling typically requires enrichment procedures that may introduce sampling bias [21,71]. Sensitivity limitations and the requirement for substantial input material remain barriers to routine clinical implementation. Moreover, reproducibility across heterogeneous tumor samples—particularly formalin-fixed paraffin-embedded (FFPE) tissues—remains an unresolved technical issue [21,72]. These limitations partly explain why non-histone methylation has remained underrepresented in clinical biomarker studies compared with phosphorylation-based signaling readouts.

### 6.2. Dynamic Versus Static Assessment of Regulatory States

Beyond technical sensitivity, a conceptual limitation concerns the distinction between static abundance and dynamic regulatory flux. Most current assays measure steady-state methylation levels, providing a snapshot of modification presence rather than functional activity. However, the framework proposed in this Review emphasizes protein methylation as a regulatory logic layer that modulates activation thresholds, signal persistence, and network topology.

Such regulatory behavior may not be fully captured by static measurements alone. For example, methylation-dependent stabilization of signaling intermediates or modulation of transcriptional persistence may operate within specific temporal windows that are not reflected in bulk steady-state levels [12,14,44,58]. Perturbation-based profiling, longitudinal sampling, or integrated multi-omic approaches may therefore be required to capture clinically relevant methylation-dependent regulatory states. Network-level modeling studies further support the view that signaling outputs emerge from combinatorial and time-dependent modification patterns rather than isolated steady-state measurements [12,73].

This dynamic-versus-static mismatch represents a key translational bottleneck: measuring modification presence does not necessarily equate to measuring regulatory consequence.

### 6.3. Toward Clinically Actionable Biomarker Integration

The clinical deployment of methylation-based biomarkers faces additional challenges. Standardized quantification thresholds have not been established for most non-histone methylation sites, and inter-patient variability in expression of methyltransferases and demethylases complicates interpretation [20,24]. Furthermore, functional cutoffs that define actionable regulatory states remain largely undefined.

Given the integrative nature of methylation-dependent signaling modulation, composite biomarker strategies may be required. Rather than relying on isolated methylation marks, clinically informative signatures may combine methylation profiling with phosphorylation readouts, transcriptional programs, or pathway activity metrics [1,7,14]. Such integrative approaches may better reflect network-level behavior and adaptive potential under therapeutic pressure [35,56].

Prospective clinical studies incorporating longitudinal sampling will be essential to determine whether methylation profiling can predict therapeutic adaptation, resistance trajectories, or combination therapy responsiveness. Until such validation is achieved, methylation measurement will remain primarily an investigative tool rather than a routine clinical assay.

### 6.4. Future Directions in Quantitative and Functional Profiling

Advances in targeted proteomics, single-cell proteomic technologies, and improved enrichment chemistries may enhance sensitivity and reproducibility in the near future [21,74,75]. Data-independent acquisition-based workflows and emerging single-cell proteomic platforms are beginning to enable deeper and more quantitative mapping of PTM landscapes across heterogeneous tumor populations [74,75]. In parallel, computational modeling approaches that integrate methylation with broader signaling networks may help infer functional regulatory states from partial datasets [12,14,73].

Ultimately, successful clinical translation will require not only improved measurement technologies but also a refined conceptual framework linking methylation-dependent regulatory logic to actionable therapeutic decisions. Bridging this gap represents a critical step toward integrating protein methylation into precision oncology.

## 7. Conclusions

Protein methylation shapes oncogenic signaling through modulation of activation thresholds, signal persistence, network topology, and spatial routing. By refining phosphorylation-driven pathways, methylation contributes to signaling heterogeneity, plasticity, and therapeutic adaptation.

Framing protein methylation as a regulatory logic layer integrates this modification into contemporary models of cancer signaling and highlights opportunities for rational therapeutic combination strategies. Rather than a secondary epigenetic modifier, protein methylation should be regarded as an integral determinant of signaling network behavior and adaptive capacity.

The framework proposed here highlights protein methylation not merely as an additional modification but as a regulatory layer that helps explain how similar kinase-driven inputs can generate diverse signaling outcomes in cancer cells.

## Figures and Tables

**Table 1:** Functional consequences of non-histone protein methylation in oncogenic signaling.

Regulatory Dimension	Functional Consequence	Representative Protein/Axis (e.g.)	Oncogenic Implication	Key Reference
Threshold/Gain Control	Altered transcriptional competence; modulation of activation sensitivity	p53 (stress-responsive transcription factor)	Bias toward cell cycle arrest, apoptosis, or adaptive survival under comparable upstream stress	[15,16,27,28]
Signal Persistence	Increased protein stability or prolonged transcriptional program maintenance	AKT–mTOR pathway node (e.g., AKT or FOXO)	Sustained proliferative or survival signaling despite fluctuating upstream activation	[30,44]
Network Topology/Complex Assembly	Modified scaffold formation and interaction interface remodeling	DDR mediator/scaffold (e.g., 53BP1 or MDC1)	Altered DNA repair complex assembly and checkpoint robustness	[33,34,45]
Spatial Routing/Subcellular Localization	Changes in nuclear–cytoplasmic distribution or receptor stability	Hormone receptor (e.g., ERα or AR)	Context-specific transcriptional routing and lineage-dependent signaling output	[46,47]

**Table 3:** Clinical development of protein methylation-targeted therapies in cancer.

Target	Representative Agent	Clinical Phase	Cancer Context	Combination Strategies	Translational Rationale	Key Reference
PRMT5	GSK3326595 (Pemrametostat)	Phase I–II	Solid tumors, lymphoma	Immune checkpoint inhibitors; DDR inhibitors	Modulation of transcriptional programs and DNA damage response; synthetic vulnerability in MTAP-deleted tumors	[39,42]
PRMT5	JNJ-64619178	Phase I	Advanced solid tumors	Monotherapy (dose escalation); combination exploration	Disruption of spliceosome regulation and signaling mediator stability	[40,42]
PRMT5	PF-06939999	Phase I	Advanced malignancies	Early-phase combination studies	Targeting arginine methylation-dependent survival pathways	[43]
Type I PRMTs (PRMT1/3/4/6)	MS023 (tool compound; preclinical)	Preclinical	Multiple tumor models	Preclinical combination with kinase inhibitors	Perturbation of transcriptional amplification and signaling feedback loops	[69]
SMYD family (e.g., SMYD3)	Experimental inhibitors	Preclinical	KRAS-driven models; breast cancer	Preclinical kinase inhibitor combinations	Modulation of MAPK/AKT axis and transcription factor methylation	[49]

## Data Availability

No new data were created or analyzed in this study. Data sharing is not applicable to this article.

## Abbreviations

AKT	Protein kinase B
AR	Androgen receptor
ATM	Ataxia telangiectasia mutated
CDK	Cyclin-dependent kinase
DDR	DNA damage response
DNA	Deoxyribonucleic acid
ERα	Estrogen receptor alpha
FFPE	Formalin-fixed paraffin-embedded
IKKβ	IκB kinase beta
JAK	Janus kinase
MAPK	Mitogen-activated protein kinase
MDC1	Mediator of DNA damage checkpoint protein 1
MTAP	Methylthioadenosine phosphorylase
mTOR	Mechanistic target of rapamycin
NF-κB	Nuclear factor kappa-light-chain-enhancer of activated B cells
PI3K	Phosphoinositide 3-kinase
PRMT	Protein arginine methyltransferase
PTM	Post-translational modification
RB	Retinoblastoma protein
SET7/9	SET domain-containing lysine methyltransferase 7/9
SMYD	SET and MYND domain-containing protein
STAT	Signal transducer and activator of transcription
TAD	Transactivation domain

## References

[1] Brognard J., Hunter T. (2011). Protein kinase signaling networks in cancer. Curr. Opin. Genet. Dev..

[2] Hunter T. (2000). Signaling—2000 and beyond. Cell.

[3] Tomuleasa C., Tigu A.B., Munteanu R., Moldovan C.S., Kegyes D., Onaciu A., Gulei D., Ghiaur G., Einsele H., Croce C.M. (2024). Therapeutic advances of targeting receptor tyrosine kinases in cancer. Signal Transduct. Target. Ther..

[4] Glaviano A., Foo A.S.C., Lam H.Y., Yap K.C.H., Jacot W., Jones R.H., Eng H., Nair M.G., Makvandi P., Geoerger B. (2023). PI3K/AKT/mTOR signaling transduction pathway and targeted therapies in cancer. Mol. Cancer.

[5] Hoxhaj G., Manning B.D. (2020). The PI3K-AKT network at the interface of oncogenic signalling and cancer metabolism. Nat. Rev. Cancer.

[6] Pottier C., Fresnais M., Gilon M., Jérusalem G., Longuespée R., Sounni N.E. (2020). Tyrosine kinase inhibitors in cancer: Breakthrough and challenges of targeted therapy. Cancers.

[7] Holohan C., Van Schaeybroeck S., Longley D.B., Johnston P.G. (2013). Cancer drug resistance: An evolving paradigm. Nat. Rev. Cancer.

[8] Garraway L.A., Jänne P.A. (2012). Circumventing cancer drug resistance in the era of personalized medicine. Cancer Discov..

[9] Hanahan D., Weinberg R.A. (2011). Hallmarks of cancer: The next generation. Cell.

[10] Yaffe M.B. (2019). Why geneticists stole cancer research even though cancer is primarily a signaling disease. Sci. Signal..

[11] Ferrell J.E., Machleder E.M. (1998). The biochemical basis of an all-or-none cell fate switch in Xenopus oocytes. Science.

[12] Bhalla U.S., Iyengar R. (1999). Emergent properties of networks of biological signaling pathways. Science.

[13] Marshall C.J. (1995). Specificity of receptor tyrosine kinase signaling: Transient versus sustained extracellular signal-regulated kinase activation. Cell.

[14] Purvis J.E., Lahav G. (2013). Encoding and decoding cellular information through signaling dynamics. Cell.

[15] Biggar K.K., Li S.S. (2015). Non-histone protein methylation as a regulator of cellular signalling and function. Nat. Rev. Mol. Cell Biol..

[16] Xu J., Richard S. (2021). Cellular pathways influenced by protein arginine methylation: Implications for cancer. Mol. Cell.

[17] Oudhoff M.J., Freeman S.A., Couzens A.L., Antignano F., Kuznetsova E., Min P.H., Northrop J.P., Lehnertz B., Barsyte-Lovejoy D., Vedadi M. (2013). Control of the hippo pathway by Set7-dependent methylation of Yap. Dev. Cell.

[18] Black J.C., Van Rechem C., Whetstine J.R. (2012). Histone lysine methylation dynamics: Establishment, regulation, and biological impact. Mol. Cell.

[19] Bedford M.T., Clarke S.G. (2009). Protein arginine methylation in mammals: Who, what, and why. Mol. Cell.

[20] Yang Y., Bedford M.T. (2013). Protein arginine methyltransferases and cancer. Nat. Rev. Cancer.

[21] Carlson S.M., Gozani O. (2014). Emerging technologies to map the protein methylome. J. Mol. Biol..

[22] Johnson L.N., Lewis R.J. (2001). Structural basis for control by phosphorylation. Chem. Rev..

[23] Bedford M.T., Richard S. (2005). Arginine methylation an emerging regulator of protein function. Mol. Cell.

[24] Hamamoto R., Nakamura Y. (2016). Dysregulation of protein methyltransferases in human cancer: An emerging target class for anticancer therapy. Cancer Sci..

[25] Chuikov S., Kurash J.K., Wilson J.R., Xiao B., Justin N., Ivanov G.S., McKinney K., Tempst P., Prives C., Gamblin S.J. (2004). Regulation of p53 activity through lysine methylation. Nature.

[26] Huang J., Perez-Burgos L., Placek B.J., Sengupta R., Richter M., Dorsey J.A., Kubicek S., Opravil S., Jenuwein T., Berger S.L. (2006). Repression of p53 activity by Smyd2-mediated methylation. Nature.

[27] Kurash J.K., Lei H., Shen Q., Marston W.L., Granda B.W., Fan H., Wall D., Li E., Gaudet F. (2008). Methylation of p53 by Set7/9 mediates p53 acetylation and activity in vivo. Mol. Cell.

[28] Shi X., Kachirskaia I., Yamaguchi H., West L.E., Wen H., Wang E.W., Dutta S., Appella E., Gozani O. (2007). Modulation of p53 function by SET8-mediated methylation at lysine 382. Mol. Cell.

[29] Lu T., Jackson M.W., Wang B., Yang M., Chance M.R., Miyagi M., Gudkov A.V., Stark G.R. (2010). Regulation of NF-kappaB by NSD1/FBXL11-dependent reversible lysine methylation of p65. Proc. Natl. Acad. Sci. USA.

[30] Yamagata K., Daitoku H., Takahashi Y., Namiki K., Hisatake K., Kako K., Mukai H., Kasuya Y., Fukamizu A. (2008). Arginine methylation of FOXO transcription factors inhibits their phosphorylation by Akt. Mol. Cell.

[31] Dasgupta M., Dermawan J.K., Willard B., Stark G.R. (2015). STAT3-driven transcription depends upon the dimethylation of K49 by EZH2. Proc. Natl. Acad. Sci. USA.

[32] Lee J.S., Smith E., Shilatifard A. (2010). The language of histone crosstalk. Cell.

[33] Carr S.M., Munro S., Kessler B., Oppermann U., La Thangue N.B. (2011). Interplay between lysine methylation and Cdk phosphorylation in growth control by the retinoblastoma protein. EMBO J..

[34] Hunter T. (2007). The age of crosstalk: Phosphorylation, ubiquitination, and beyond. Mol. Cell.

[35] Sharma S.V., Lee D.Y., Li B., Quinlan M.P., Takahashi F., Maheswaran S., McDermott U., Azizian N., Zou L., Fischbach M.A. (2010). A chromatin-mediated reversible drug-tolerant state in cancer cell subpopulations. Cell.

[36] Konieczkowski D.J., Johannessen C.M., Garraway L.A. (2018). A Convergence-Based Framework for Cancer Drug Resistance. Cancer Cell.

[37] Kim K.H., Roberts C.W. (2016). Targeting EZH2 in cancer. Nat. Med..

[38] Kim H., Ronai Z.A. (2020). PRMT5 function and targeting in cancer. Cell Stress.

[39] Chan-Penebre E., Kuplast K.G., Majer C.R., Boriack-Sjodin P.A., Wigle T.J., Johnston L.D., Rioux N., Munchhof M.J., Jin L., Jacques S.L. (2015). A selective inhibitor of PRMT5 with in vivo and in vitro potency in MCL models. Nat. Chem. Biol..

[40] Brehmer D., Beke L., Wu T., Millar H.J., Moy C., Sun W., Mannens G., Pande V., Boeckx A., van Heerde E. (2021). Discovery and Pharmacological Characterization of JNJ-64619178, a Novel Small-Molecule Inhibitor of PRMT5 with Potent Antitumor Activity. Mol. Cancer Ther..

[41] Kryukov G.V., Wilson F.H., Ruth J.R., Paulk J., Tsherniak A., Marlow S.E., Vazquez F., Weir B.A., Fitzgerald M.E., Tanaka M. (2016). MTAP deletion confers enhanced dependency on the PRMT5 arginine methyltransferase in cancer cells. Science.

[42] Vieito M., Moreno V., Spreafico A., Brana I., Wang J.S., Preis M., Hernández T., Genta S., Hansen A.R., Doger B. (2023). Phase 1 study of JNJ-64619178, a protein arginine methyltransferase 5 inhibitor, in advanced solid tumors. Clin. Cancer Res..

[43] Rodon J., Rodriguez E., Maitland M.L., Tsai F.Y., Socinski M.A., Berlin J.D., Thomas J.S., Al Baghdadi T., Wang I.M., Guo C. (2024). A phase I study to evaluate the safety, pharmacokinetics, and pharmacodynamics of PF-06939999 (PRMT5 inhibitor) in patients with selected advanced or metastatic tumors with high incidence of splicing factor gene mutations. ESMO Open.

[44] Guo J., Dai X., Laurent B., Zheng N., Gan W., Zhang J., Guo A., Yuan M., Liu P., Asara J.M. (2019). AKT methylation by SETDB1 promotes AKT kinase activity and oncogenic functions. Nat. Cell Biol..

[45] Boisvert F.M., Rhie A., Richard S., Doherty A.J. (2005). The GAR motif of 53BP1 is arginine methylated by PRMT1 and is necessary for 53BP1 DNA binding activity. Cell Cycle.

[46] Le Romancer M., Treilleux I., Leconte N., Robin-Lespinasse Y., Sentis S., Bouchekioua-Bouzaghou K., Goddard S., Gobert-Gosse S., Corbo L. (2008). Regulation of estrogen rapid signaling through arginine methylation by PRMT1. Mol. Cell.

[47] Subramanian K., Jia D., Kapoor-Vazirani P., Powell D.R., Collins R.E., Sharma D., Peng J., Cheng X., Vertino P.M. (2008). Regulation of estrogen receptor alpha by the SET7 lysine methyltransferase. Mol. Cell.

[48] Saddic L.A., West L.E., Aslanian A., Yates J.R., Rubin S.M., Gozani O., Sage J. (2010). Methylation of the retinoblastoma tumor suppressor by SMYD2. J. Biol. Chem..

[49] Hamamoto R., Furukawa Y., Morita M., Iimura Y., Silva F.P., Li M., Yagyu R., Nakamura Y. (2004). SMYD3 encodes a histone methyltransferase involved in the proliferation of cancer cells. Nat. Cell Biol..

[50] Yang X.D., Tajkhorshid E., Chen L.F. (2010). Functional interplay between acetylation and methylation of the RelA subunit of NF-kappaB. Mol. Cell Biol..

[51] Hayden M.S., Ghosh S. (2011). NF-κB in immunobiology. Cell Res..

[52] Yu H., Pardoll D., Jove R. (2009). STATs in cancer inflammation and immunity: A leading role for STAT3. Nat. Rev. Cancer.

[53] Komyod W., Bauer U.M., Heinrich P.C., Haan S., Behrmann I. (2005). Are STATS arginine-methylated?. J. Biol. Chem..

[54] Ali S., Coombes R.C. (2000). Estrogen receptor alpha in human breast cancer: Occurrence and significance. J. Mammary Gland. Biol. Neoplasia.

[55] Ahn S., Park J.H., Grimm S.L., Piyarathna D.W.B., Samanta T., Putluri V., Mezquita D., Fuqua S.A.W., Putluri N., Coarfa C. (2024). Metabolomic Rewiring Promotes Endocrine Therapy Resistance in Breast Cancer. Cancer Res..

[56] Gerlinger M., Rowan A.J., Horswell S., Math M., Larkin J., Endesfelder D., Gronroos E., Martinez P., Matthews N., Stewart A. (2012). Intratumor heterogeneity and branched evolution revealed by multiregion sequencing. N. Engl. J. Med..

[57] Zhang J., Yu Y., Zou X., Du Y., Liang Q., Gong M., He Y., Luo J., Wu D., Jiang X. (2024). WSB1/2 target chromatin-bound lysine-methylated RelA for proteasomal degradation and NF-κB termination. Nucleic Acids Res..

[58] Yang J., Huang J., Dasgupta M., Sears N., Miyagi M., Wang B., Chance M.R., Chen X., Du Y., Wang Y. (2010). Reversible methylation of promoter-bound STAT3 by histone-modifying enzymes. Proc. Natl. Acad. Sci. USA.

[59] Hata A.N., Niederst M.J., Archibald H.L., Gomez-Caraballo M., Siddiqui F.M., Mulvey H.E., Maruvka Y.E., Ji F., Bhang H.E., Krishnamurthy Radhakrishna V. (2016). Tumor cells can follow distinct evolutionary paths to become resistant to epidermal growth factor receptor inhibition. Nat. Med..

[60] Chong C.R., Jänne P.A. (2013). The quest to overcome resistance to EGFR-targeted therapies in cancer. Nat. Med..

[61] Engelman J.A., Zejnullahu K., Mitsudomi T., Song Y., Hyland C., Park J.O., Lindeman N., Gale C.M., Zhao X., Christensen J. (2007). MET amplification leads to gefitinib resistance in lung cancer by activating ERBB3 signaling. Science.

[62] Prahallad A., Sun C., Huang S., Di Nicolantonio F., Salazar R., Zecchin D., Beijersbergen R.L., Bardelli A., Bernards R. (2012). Unresponsiveness of colon cancer to BRAF(V600E) inhibition through feedback activation of EGFR. Nature.

[63] Nazarian R., Shi H., Wang Q., Kong X., Koya R.C., Lee H., Chen Z., Lee M.K., Attar N., Sazegar H. (2010). Melanomas acquire resistance to B-RAF(V600E) inhibition by RTK or N-RAS upregulation. Nature.

[64] Newman A.C., Maddocks O.D.K. (2017). One-carbon metabolism in cancer. Br. J. Cancer.

[65] Mentch S.J., Locasale J.W. (2016). One-carbon metabolism and epigenetics: Understanding the specificity. Ann. N. Y. Acad. Sci..

[66] Ducker G.S., Rabinowitz J.D. (2017). One-Carbon Metabolism in health and disease. Cell Metab..

[67] Shaffer S.M., Dunagin M.C., Torborg S.R., Torre E.A., Emert B., Krepler C., Beqiri M., Sproesser K., Brafford P.A., Xiao M. (2017). Rare cell variability and drug-induced reprogramming as a mode of cancer drug resistance. Nature.

[68] Hangauer M.J., Viswanathan V.S., Ryan M.J., Bole D., Eaton J.K., Matov A., Galeas J., Dhruv H.D., Berens M.E., Schreiber S.L. (2017). Drug-tolerant persister cancer cells are vulnerable to GPX4 inhibition. Nature.

[69] Eram M.S., Shen Y., Szewczyk M., Wu H., Senisterra G., Li F., Butler K.V., Kaniskan H.Ü., Speed B.A., Dela Seña C. (2016). A Potent, Selective, and Cell-Active Inhibitor of Human Type I Protein Arginine Methyltransferases. ACS Chem. Biol..

[70] Mavrakis K.J., McDonald E.R., Schlabach M.R., Billy E., Hoffman G.R., deWeck A., Ruddy D.A., Venkatesan K., Yu J., McAllister G. (2016). Disordered methionine metabolism in MTAP/CDKN2A-deleted cancers leads to dependence on PRMT5. Science.

[71] Carlson S.M., Moore K.E., Green E.M., Martín G.M., Gozani O. (2014). Proteome-wide enrichment of proteins modified by lysine methylation. Nat. Protoc..

[72] Azimzadeh O., Barjaktarovic Z., Aubele M., Calzada-Wack J., Sarioglu H., Atkinson M.J., Tapio S. (2010). Formalin-fixed paraffin-embedded (FFPE) proteome analysis using gel-free and gel-based proteomics. J. Proteome Res..

[73] Invergo B.M., Beltrao P. (2018). Reconstructing phosphorylation signalling networks from quantitative phosphoproteomic data. Essays Biochem..

[74] Bekker-Jensen D.B., Bernhardt O.M., Hogrebe A., Martinez-Val A., Verbeke L., Gandhi T., Kelstrup C.D., Reiter L., Olsen J.V. (2020). Rapid and site-specific deep phosphoproteome profiling by data-independent acquisition without the need for spectral libraries. Nat. Commun..

[75] Budnik B., Levy E., Harmange G., Slavov N. (2018). SCoPE-MS: Mass spectrometry of single mammalian cells quantifies proteome heterogeneity during cell differentiation. Genome Biol..

